# Involvement of Serotonergic System in Oxaliplatin-Induced Neuropathic Pain

**DOI:** 10.3390/biomedicines9080970

**Published:** 2021-08-06

**Authors:** Ji Hwan Lee, Woojin Kim

**Affiliations:** Department of Physiology, College of Korean Medicine, Kyung Hee University, Seoul 02453, Korea; mibdna@khu.ac.kr

**Keywords:** allodynia, chemotherapy-induced peripheral neuropathic pain, oxaliplatin, serotonin

## Abstract

Oxaliplatin is a chemotherapeutic agent widely used against colorectal and breast cancers; however, it can also induce peripheral neuropathy that can rapidly occur even after a single infusion in up to 80–90% of treated patients. Numerous efforts have been made to understand the underlying mechanism and find an effective therapeutic agent that could diminish pain without damaging its anti-tumor effect. However, its mechanism is not yet clearly understood. The serotonergic system, as part of the descending pain inhibitory system, has been reported to be involved in different types of pain. The malfunction of serotonin (5-hydroxytryptamine; 5-HT) or its receptors has been associated with the development and maintenance of pain. However, its role in oxaliplatin-induced neuropathy has not been clearly elucidated. In this review, 16 in vivo studies focused on the role of the serotonergic system in oxaliplatin-induced neuropathic pain were analyzed. Five studies analyzed the involvement of 5-HT, while fourteen studies observed the role of its receptors in oxaliplatin-induced allodynia. The results show that 5-HT is not involved in the development of oxaliplatin-induced allodynia, but increasing the activity of the 5-HT_1A_, 5-HT_2A_, and 5-HT_3_ receptors and decreasing the action of 5-HT_2C_ and 5-HT_6_ receptors may help inhibit pain.

## 1. Introduction

Oxaliplatin is a third-generation platinum-based chemotherapeutic drug widely used to treat various types of cancer [1,2,3]. Although it is effective against tumors, it can also induce side effects, such as mouth soreness, nausea, and vomiting, which may limit its use [4,5]. Among the various negative effects, the most prevalent is peripheral neuropathy, which is expressed as cold and mechanical allodynia in the feet and hands [6,7,8]. Dysesthesia and paresthesia can occur as early as 48 h after infusion [8]. Numerous studies have been conducted to understand the underlying mechanisms, and although it is yet clearly understood, malfunction of voltage-gated sodium channels [9] and organic cation transporters [10], mitochondrial dysfunction [11], oxidative stress [12], axonal degeneration [13], and impairment of the descending pain inhibitory system [14] have been proposed as the causes of this peripheral neuropathy.

Several groups of chemotherapy substances can cause peripheral neuropathy, such as platinum-based agents, taxanes, and immunomodulatory drugs; however, among them, platinum-based drugs are reported to cause the highest rate of peripheral neuropathy (70–100%) compared to other agents (e.g., taxanes and immunomodulatory drugs, 11–87% and 20–60%, respectively). Among the three platinum-based drugs (oxaliplatin, cisplatin, and carboplatin), acute neuropathy develops in approximately 65–98% of patients within hours of oxaliplatin infusion at a dose ranging from 85 to 130 mg/m^2^, whereas cisplatin is induced in 92% of patients after cumulative treatment (500–600 mg/m^2^), and carboplatin is less toxic, as 13–42% of patients are induced [15,16,17]. These results showed that oxaliplatin could acutely induce neuropathic pain with higher rate compared to other platinum-based drugs.

For many years, our lab has focused on oxaliplatin-induced neuropathic pain, and continuous efforts have been made to understand its pathophysiology and to find an effective treatment that could reduce pain without affecting its anti-tumor effects [18,19,20,21,22,23,24]. Among many pathways, the serotonergic system in the central nervous system (CNS), which is part of the descending pain inhibitory system, has been shown to be deeply involved in oxaliplatin-induced neuropathic pain [25,26,27,28,29].

Serotonin (5-hydroxytryptamine; 5-HT) is a monoaminergic neurotransmitter synthesized from tryptophan via the sequential actions of tryptophan hydroxylase. In the CNS, it is produced primarily in the brainstem (rostro ventromedial medulla; RVM) [30], and in the peripheral nervous system (PNS), the main cellular sources of 5-HT are platelets and mast cells [31,32].

Synthesized 5-HT can interact with seven different families of 5-HT receptors that comprise 15 subtypes [30,33]. Among the seven classes, six are G-protein coupled receptors (5-HT_1,2,4-7_ receptors), whereas one is a ligand-gated cation channel (5-HT_3_ receptor) [34]. 5-HT and its receptors are widely known to be involved in pain attenuation [35,36], and enhancing 5-HT [37,38] or modulating the function of its receptor in the spinal cord has been reported to decrease pain in various animal models [35,39]. Among 5-HT receptor subtypes, 5-HT_1A_, 5-HT_6_ and 5-HT_7_ receptors are coupled to Gi/o, suggesting an inhibitory effect [40,41,42,43]. In contrast 5-HT_2_ receptor is coupled to Gq/11, and 5-HT_3_ receptor is directly linked to non-selective cationic channels, suggesting an excitatory effect [40,44,45]. However, their effect in pain is known to vary according to the types of pain and experimental conditions [35].

In our previous study [46], spinal mRNA expression of 5-HT_1A_ receptors was downregulated 4 days after oxaliplatin injection, when pain behaviors were obvious, and it was upregulated when cold and mechanical allodynia were alleviated in mice. Moreover, intrathecal injection of 5-HT_1A_ (NAN-190), 5-HT_2A_ (ketanserin), or 5-HT_3_ (MDL-72222) receptor antagonists inhibited the analgesic effect of various treatments, suggesting that targeting the serotonergic system may be an effective method to modulate oxaliplatin-induced neuropathic pain [25,28].

Furthermore, although no agent has been recommended for prevention in the recently published guidelines from the American Society of Clinical Oncology (ASCO), duloxetine, a serotonin-norepinephrine reuptake inhibitor (SNRI), has been recommended for the treatment of oxaliplatin-induced neuropathic pain [47]. Duloxetine has been reported to be effective in alleviating allodynia in several clinical trials [48,49], and although duloxetine is a SNRI, it is known to more potently block 5-HT than norepinephrine (NE) transporter, showing that 5-HT play an important role in the anti-allodynic effect of duloxetine [50]. In addition, in different animal models of neuropathic pain, fluoxetine, a selective serotonin reuptake inhibitors, significantly attenuate the pain, demonstrating the analgesic effect of 5-HT [51]. However, the underlying mechanism of action of serotonergic system is not yet clearly defined.

Therefore, clarifying the role of 5-HT in oxaliplatin-induced neuropathic pain is important not only to understand pain but also to develop optimal drugs. However, to date, no reviews have been published that summarize the involvement of the serotonergic system in oxaliplatin-induced neuropathic pain. In this review, by analyzing all studies that observed the involvement of 5-HT and its receptors, we will discuss the role of 5-HT and its receptors in oxaliplatin-induced peripheral neuropathic pain.

## 2. Results

### 2.1. Role of 5-HT in Oxaliplatin-Induced Neuropathic Pain

Five studies investigated the role of monoamine neurotransmitters in oxaliplatin-induced neuropathic pain (Table 1). Among many neurotransmitters, it is well accepted that 5-HT is involved in the pain modulation, although 5-HT is known to exert both pain faciliatory and inhibitory effect depending on the pain states and the type of receptors [32,35]. However, experimental studies reported that direct application of 5-HT into the spinal cord generally inhibited nociceptive responses [52]. This may be due to the fact that 5-HT produce excitatory effect on many inhibitory neurons such as GABA (gamma-Aminobutyric acid) and glycine present in the spinal dorsal horn [39,53]. In this review, four studies conducted experiments by depleting 5-HT using PCPA [27,28,29,54], while one study directly measured the level of 5-HT in several areas of the brain [55]. PCPA is an irreversible inhibitor of tryptophan hydroxylase, which is used to synthesize 5-HT from tryptophan [56]. PCPA pretreatment is generally used to deplete 5-HT and has been reported to reduce central and peripheral 5-HT [57]. PCPA decreased the levels of 5-HT and its metabolite 5-hydroxyindoleacetic acid to 9.4% and 8.2% of control levels, respectively, in rats, without affecting the levels of norepinephrine and dopamine [58].

Masuguchi et al. [54] reported that 5-HT depletion did not aggravate or prevent the development of cold and mechanical allodynia in rats. Three times intraperitoneal injection of PCPA significantly reduced the 5-HT content by 63% in the spinal cord (L1-L6, 519.1 ± 16.8 ng/g vs. 189.5 ± 22.2 ng/g), but oxaliplatin-induced neuropathic pain remained unaffected. However, depletion of 5-HT reduced the analgesic effect of neurotropin, as its analgesic effect was abolished after 5-HT depletion. Lee et al. [27] also used PCPA to deplete 5-HT in rats. PCPA was injected for 3 days and oxaliplatin was administered on the last day. Their results showed that PCPA depletion did not affect the development of oxaliplatin-induced cold allodynia. However, PCPA prevented the analgesic effect induced by subcutaneous injection of 0.25 mg/kg of bee venom acupuncture (BVA) at the GV3 acupuncture point. In their study, BVA administration significantly increased the level of 5-HT in the spinal cord, showing that increased 5-HT concentration resulted in oxaliplatin-induced pain attenuation. Similar to the results of Lee et al., Li et al. [29] also demonstrated that 5-HT depletion did not influence the development of cold and mechanical allodynia in mice. In their study, bee venom-derived phospholipase A_2_ (bvPLA_2_), which is one of the major subcomponents of BVA, demonstrated analgesic effects against both cold and mechanical allodynia even after PCPA injection, showing that the development and treatment were not affected by 5-HT. In a study by Li et al. [28], 5-HT depletion did not prevent or enhance pain development in mice; however, the analgesic effect of venlafaxine, an SNRI, on mechanical but not cold allodynia, was significantly blocked by pretreatment with PCPA.

Although Hache et al. [55] did not use PCPA to observe the effect of 5-HT on oxaliplatin-induced neuropathic pain, but they measured the 5-HT content in the anterior cingulate cortex (ACC) of mice before and after the administration of various monoamine reuptake inhibitors, such as SNRI, serotonin reuptake inhibitor, and triple reuptake inhibitors. ACC is an area of the brain known to be important for pain-related perception [59,60]. It receives dense 5-HT and norepinephrine innervation, and its descending projections are reported to be transmitted to RVM neurons [61]. Hache et al. conducted von Frey, cold plate, and thermal preference plate tests to assess its effect on oxaliplatin-induced mechanical, cold, and thermal allodynia. In the von Frey hair test, all drugs, except for venlafaxine, significantly decreased oxaliplatin-induced allodynia. In the cold plate test, only indatraline significantly increased the latency of the first jump compared to the control. In the thermal preference test, all drugs, but not escitalopram, were significantly effective. All four drugs significantly elevated the dose of 5-HT in ACC, but escitalopram and venlafaxine induced more extracellular 5-HT levels than norepinephrine, whereas indatraline and NS18283 increased norepinephrine levels compared to 5-HT.

### 2.2. Role of 5-HT Receptors in Oxaliplatin-Induced Neuropathic Pain

In total, 14 studies analyzed the involvement of 5-HT receptors in oxaliplatin-induced neuropathic pain. Nine studies focused on 5-HT_1_ [26,27,46,54,62,63,64,65,66], six focused on 5-HT_2_ [26,27,54,65,67,68], five on 5-HT_3_ [25,26,27,28,54], and one study focused on 5-HT_6_ [69] receptors. Most of the studies focused on the spinal cord, but two studies observed changes in the brain [67] and skin [63] (Table 2).

#### 2.2.1. 5-HT_1_ Receptors

Five different subtypes of 5-HT_1_ receptors are present (5-HT_1A, B, D, E, and F_) [70]_._ Although the role of 5-HT_1D, E, and F_ receptors in the pain state is poorly understood [71], 5-HT_1A_ and B receptor agonists have been reported to reduce pain [72]. In this study, all nine studies focused on 5-HT_1A_ receptors; 5-HT_1A_ receptors are involved in pain attenuation, since spinal 5-HT_1A_ receptor stimulation using selective agonists resulted in analgesia in neuropathic animals [73,74]. In the CNS, a large number of 5-HT_1A_ receptors are present in serotonergic cells, mainly in the dorsal and median raphe nuclei [35]. In the spinal cord, 5-HT_1A_ receptors are known to be present in GABAergic interneurons located in the superficial and deeper layers of the spinal dorsal horn [75]. In the PNS, they are expressed in capsaicin-sensitive C-fibers [63,76].

Andoh et al. [62] reported that oxaliplatin produced mechanical allodynia from D7 to D14 and that the mRNA level of 5-HT_1A_ receptors in the spinal dorsal horn, but not in the DRG, increased after a single injection of oxaliplatin. Although a single oral treatment with xaliproden, a selective 5-HT_1A_ receptor agonist, failed to suppress oxaliplatin-induced mechanical allodynia, xaliproden partially suppressed the increase in tibial nerve firing after oxaliplatin treatment. The nerve firing was evoked with 0.69 mN of von Frey filament. However, this inhibition (32%) was not significant enough to attenuate mechanical allodynia. In their study, xaliproden significantly attenuated mechanical allodynia induced by paclitaxel injection, which is another chemotherapeutic agent, and the nerve response decreased to 68% compared to paclitaxel-treated mice. On the contrary to the study of Andoh et al., in a study by Lee et al. [46] mRNA level of spinal 5-HT_1A_ receptors was significantly downregulated after oxaliplatin treatment and upregulated when allodynic signs were alleviated. This discordance may be due to the difference in measurement time and the dose of oxaliplatin, as Andoh et al. observed 10 days after 3 mg/kg of oxaliplatin injection, while Lee et al. conducted experiments 5 days after 6 mg/kg of oxaliplatin. Due to the limited number of studies that observed the mRNA expression of 5-HT_1A_ in the spinal cord after oxaliplatin treatment, it is hard to draw a firm conclusion; however, as in the spinal cord, 5-HT_1A_ receptors are known to be expressed both in inhibitory interneurons and non-inhibitory interneurons [77,78], future study should be conducted to clarify which 5-HT_1A_ receptors are upregulated or downregulated after oxaliplatin treatment.

In another study conducted by Andoh et al. [63] daily oral treatment with selective 5-HT_1A_ receptor agonists (xaliproden or tandospirone) for 10 days significantly prevented the development of oxaliplatin-induced mechanical allodynia in mice. Moreover, treatment with selective 5-HT_1A_ receptor agonists significantly reduced the number of mast cells in the plantar skin of mice. These results suggested that 5-HT_1A_ receptor agonists may decrease oxaliplatin-induced mast cell migration by inhibiting the release of substance P from C-fiber afferent neurons. It should be noted that activation of the 5-HT_1A_ receptor has been reported to cause hyperpolarization of capsaicin-sensitive neurons [79,80]. Mast cells are known to participate in pain development by releasing inflammatory mediators such as ATP, histamine, and tryptase [81]. Moreover, Sakamoto et al. [82] have reported that oxaliplatin may activate C-fiber to release neuromodulators, which could degranulates mast cells. Subsequently, tryptase released from the mast cell can sensitize the A-fibers which could lead to allodynia.

According to the studies of Salat et al. and Rapacz et al. [64,65], intraperitoneal injection of NLX-112 and JOA 112 (3p), attenuated oxaliplatin-induced mechanical allodynia at the acute and late phase (3 h and 7 days after the injection of oxaliplatin, respectively). NLX-112 is a selective 5-HT_1A_ receptor agonist, while JOA 112 (3p) has a moderate affinity for the 5-HT_1A_ receptors (K_i_ ± SEM, 223.0 ± 4.5 nM). These results suggest that intraperitoneal injection of 5-HT_1A_ receptor agonist may participate in the suppression of oxaliplatin-induced mechanical allodynia. In accordance with those results, Panczyk et al. [66] reported that administration of 1-[3-(2,4,6-trimethylphenoxy)propyl]-4-(4-methoxyphenyl) piperazine dihydrochloride, which has the potency of a 5-HT_1A_ receptor antagonist, did not show any significant analgesic effects against oxaliplatin-induced cold and mechanical allodynia.

Furthermore, by using a potent 5-HT_1A_ antagonist WAY100635, Masuguchi et al. [54] reported that neurotropin (100 and 200 NU/kg) could significantly attenuate cold allodynia via the action of spinal 5-HT_1A_ receptors, as WAY100635 pretreatment blocked the effect of neurotropin against cold allodynia.

Another potent 5-HT_1A_ receptor antagonist (NAN-190) was used in two other studies conducted with BVA [27] and electroacupuncture (EA) [26]. In their experiments, intrathecal injection of 5-HT_1A_ receptor antagonist failed to inhibit the anti-allodynic effect of BVA and EA administered at Yaoyangguan (GV3) and Zusanli (ST36) acupuncture point, respectively, indicating that the effect of BVA and EA was not mediated by spinal 5-HT_1A_ receptors. However, although intrathecal treatment of 5-HT_1A_ receptors antagonist failed to inhibit the effect of BVA, when mixed 5-HT_1/2_ antagonists (methysergide) was pretreated intraperitoneally the effect of BVA was blocked showing that the effect of BVA may be mostly mediated by peripheral 5-HT_1_ or 5-HT_2_ receptors than 5-HT_1A_ receptors in the spinal cord.

#### 2.2.2. 5-HT_2_ Receptors

The 5-HT_2_ receptors are G-protein coupled receptors, with three subtypes, 5-HT_2A_, 5-HT_2B_, and 5-HT_2C_. Compared to 5-HT_1_ receptors, 5-HT_2_ receptors are present in the spinal cord with relatively low density [83], and they are primarily reported to be found in the ventral than in the dorsal horn of the spinal cord [84]. The 5-HT_2A_ and 5-HT_2C_ receptors have a widespread distribution and function in the CNS, whereas 5-HT_2B_ receptors have restricted expression [85]. In the included studies, the roles of 5-HT_2A_ and 5-HT_2C_ receptors were observed.

The involvement of 5-HT_2A_ receptors in cold and mechanical allodynia has been assessed by Masuguchi et al. [54]. Intrathecal administration of 5-HT_2A_ receptor antagonists (ketanserin) significantly inhibited the effect of neurotropin on cold hyperalgesia and mechanical allodynia, suggesting that spinal 5-HT_2A_ receptor activation may lead to oxaliplatin-induced neuropathic pain attenuation. However, in the studies by Lee et al. [27] and Lee et al. [26] 5-HT_2A_ receptor antagonists (ketanserin) failed to block the analgesic effect of BVA and EA, demonstrating that 5-HT_2A_ receptors are not involved in their pain-alleviating pathways.

Although in this review, only one study demonstrated the involvement of 5-HT_2A_ receptors in oxaliplatin-induced allodynia, the pain suppression effect of 5-HT_2A_ receptors has been reported in different types of pain. In a mono-arthritis animal model of pain, upregulated mRNA expression of 5-HT_2A_ receptors was observed in the nucleus of the RVM, ventrolateral periaqueductal gray, and spinal cord when the pain was attenuated [86]. Moreover, in an animal model of diabetes- and traumatic-induced neuropathic pain, increase in 5-HT_2A_ receptors responsiveness resulted to pain inhibition [87,88]. In addition, in nerve injury-induced mechanical and thermal hyperalgesia, the activation of spinal 5-HT_2A_ receptor-induced upregulation of potassium chloride co-transporter type 2 (KCC2) and suppressed pain [89].

Baptista-de-Souza et al. [67] analyzed the mRNA and protein expression of 5-HT_2C_ receptors in the spinal cord, periaqueductal gray (PAG), and amygdala (AMY) after oxaliplatin and fluoxetine administration. Oxaliplatin administration induced mechanical and cold allodynia in rats and upregulated the mRNA and protein expression of 5-HT_2C_ receptors in the spinal cord and PAG but was downregulated in AMY. Multiple subcutaneous injections of fluoxetine, which acts as a competitive 5-HT_2C_ receptor antagonist, decreased mechanical and cold allodynia induced by oxaliplatin. Moreover, it decreased the mRNA and protein levels of 5-HT_2C_ receptors in the spinal cord, while in the AMY, they were increased. In PAG, only the protein, but not the mRNA level, was upregulated. In a surgical paw incision pain model rodents, blocking the spinal 5-HT_2C_ receptor prevented the hyperactivity of spinal neuron [90]. Furthermore, activation of 5-HT_2C_ receptors in the AMY has been reported to enhance fear-induced antinociception in rats [91]. Also, activation of 5-HT_2C_ receptors present in the PAG was demonstrated to increases antinociception in mice exposed to the elevated plus-maze [92]. Altogether, these result support that decreasing the activity of spinal 5-HT_2C_ receptors in the spinal cord, and increasing the function of 5-HT_2C_ receptors in the AMY and PAG could result in pain reduction.

Chenaf et al. [68] also demonstrated that intraperitoneal treatment with agomelatine, a 5-HT_2C_ receptor antagonist, significantly suppressed oxaliplatin-induced cold allodynia at 45 min and 24 h after injection. These results suggest that both 5-HT_2A_ receptor agonists and 5-HT_2C_ receptor antagonists could attenuate oxaliplatin-induced neuropathic pain.

#### 2.2.3. 5-HT_3_ Receptors

Among all 5-HT receptor families, 5-HT_3_ receptors are the only non-selective ligand-gated ion channels [93]. In the PNS, they are localized in the DRG and in the myelinated and unmyelinated primary afferent fiber terminals [94]. As ligand-gated ion channels, activation of 5-HT_3_ receptors in DRG has been shown to induce pronociceptive effects [95,96]. However, in the spinal cord, they are mostly found in the superficial laminae and inhibitory GABAergic interneurons showing antinociceptive effects [35].

Five studies have observed the role of spinal 5-HT_3_ receptors in the analgesic effect on oxaliplatin-induced pain. Although different treatment methods have been applied in each study, they all reported that intrathecal pretreatment with a 5-HT_3_ receptor antagonist (MDL-72222) significantly blocked the analgesic effect. Masuguchi et al. [54] reported that the effect of neurotropin was inhibited by a 5-HT_3_ receptor antagonist. Lee et al. [27] and Kim et al. [25] showed that the anti-allodynic effect of BVA alone or with morphine was blocked by MDL-72222 pretreatment. Lee et al. [26] and Li et al. [28] also reported that the effects of EA and venlafaxine were blocked by a 5-HT_3_ receptor antagonist. These results show that intrathecal administration of 5-HT_3_ receptor agonists may be an effective agent for treating oxaliplatin-induced neuropathic pain.

#### 2.2.4. 5-HT_6_ Receptors

5-HT_6_ receptors are one of the most recently added receptors to the 5-HT family [97]. They are expressed in the excitatory interneurons of the spinal cord dorsal horn [33]. In an animal model of spinal nerve injury, 5-HT_6_ receptor antagonists significantly blocked allodynia [98], and in the rat formalin test, both spinal and peripheral 5-HT_6_ receptors played a pronociceptive role [99].

Martin et al. [69] observed the effect of the 5-HT_6_ receptor inverse agonist and antagonist, SB258585 and PZ-1388, respectively, on oxaliplatin-induced cold and mechanical pain behavior. Both drugs significantly attenuated oxaliplatin-induced pain behavior. Furthermore, injection of an interfering peptide (Tat-VEPE), which loosens the interaction between the 5-HT_6_ receptor and mTOR, attenuates oxaliplatin-induced cold and mechanical allodynia. Notably, 5-HT_6_ receptors are known to engage in mTOR signaling, which is reported to be involved in the modulation of neuropathic pain [100].

## 3. Discussion

In this review, 16 animal studies focused on the serotonergic system in oxaliplatin-induced neuropathic pain were analyzed. Five studies [27,28,29,54,55] focused on the role of 5-HT, while fourteen studies observed the role of its receptors in oxaliplatin-induced allodynia. Three studies discussed both 5-HT and its receptors [27,28,54]. To our knowledge, this is the first review that focuses on the involvement of the serotonergic system in oxaliplatin-induced neuropathic pain. As part of the descending pain inhibitory system, the serotonergic system has long been known to play an active role in various types of pain [35,36,101,102]. However, its role in oxaliplatin-induced neuropathic pain has not been clearly defined.

**Figure 1:**
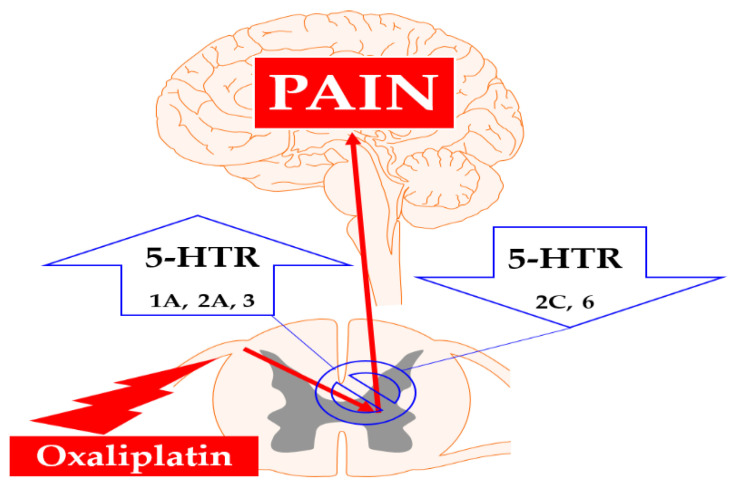
The involvement of spinal serotonergic receptors in the alleviation of oxaliplatin-induced neuropathic pain. Oxaliplatin administration increases the transmission of nociceptive signals from the primary afferent fibers to the brain (red). Activation of spinal 5-HT_1A, 2A, and 3_ receptors and inhibition of spinal 5-HT_2C and 6_ receptors could reduce allodynia induced by oxaliplatin (blue). 5-HTR; 5-HT receptors.

Among the included studies, four studies pretreated PCPA to observe the role of 5-HT in oxaliplatin-induced allodynia in rodents. Their results showed that 5-HT may not be involved in the development of neuropathic pain, as 5-HT depletion did not aggravate or attenuate oxaliplatin-induced neuropathic pain [26,28,29,54]. In contrast, 5-HT important in mediating the analgesic effect, as some drugs did not alleviate allodynia when 5-HT was depleted [27,28,54]. These results are consistent with the guidelines of ASCO as SNRI, which increases the level of monoamine neurotransmitters, has been recommended only for treatment but not for prevention of chemotherapy-induced neuropathic pain [47]. Furthermore, these results also suggest that the impairment of 5-HT in the descending pain inhibitory system may not be the leading cause of oxaliplatin-induced pain. Oxaliplatin may be more focused on altering the function of neurons in the periphery (e.g., altering the function of Na^+^ channels [9]) than in the brain or the spinal cord, as it has limited permeability to the BBB [103,104]. Thus, 5-HT concentration in the CNS and PNS may not affect the development of pain; however, 5-HT could decrease pain by modulating pain signal transmission through the action of its receptors present in the brain, spinal cord, and peripheral nerves (Figure 1). In line with this, noradrenaline depletion [28] or silencing the locus coeruleus region [105] where most of the descending noradrenergic system originate did not aggravate the oxaliplatin-induced neuropathic pain showing that both 5-HT and noradrenaline depletion does not affect the development of neuropathic pain. These results suggest that attenuating the 5-HT and noradrenergic system does not affect; however, increasing the tone of noradrenaline and 5-HT may affect the development of pain.

To observe the role of 5-HT_1A_ receptors, various types of 5-HT_1A_ receptor agonists have been administered. Xaliproden, a selective 5-HT_1A_ receptor agonist, did not attenuate oxaliplatin-induced mechanical allodynia when administered orally; however, its multiple administrations significantly reduced pain. Xaliproden was administered for 10 consecutive days and the pain alleviating effect initiated on D3 and peaked at D10 [63]. These results show that single treatment may not be sufficient to attenuate oxaliplatin-induced neuropathic pain. Although conducted in different animal model of disease, single administration of 5-HT_1A_ receptors resulted in acute decrease of 5-HT level in the brain area, whereas chronic treatment for 21 days resulted in increase of 5-HT synthesis [106], this may partially explain why multiple, but not single treatment succeeded to alleviated allodynia.

Intraperitoneal injection of NLX-112, which is reported to have full agonist activity against 5-HT_1A_ receptors, also decreased mechanical allodynia. Tandospirone is known to be a highly potent partial agonist of 5-HT_1A_ receptors, as it has a *K*_i_ value of 27 ± 5 nM [107], and its multiple treatments significantly attenuated pain such as xaliproden. Moreover, JOA 122 (3p), which has a moderate affinity with a *K*_i_ value of 223.0 ± 4.5 nM, decreased the acute and late phases of oxaliplatin-induced neuropathic pain. However, the 5-HT_1A_ receptor antagonist (compound 3), with a *K*_i_ value of 146.0 ± 28.4 nM, did not affect oxaliplatin-induced neuropathy. These results suggest that therapeutic agents that could stimulate both spinal and peripheral 5-HT_1A_ receptors can effectively alleviate oxaliplatin-induced neuropathic pain.

In contrast to 5-HT_1A_ receptors, Baptista-de-Souza et al. [67] and Chenaf et al. [68] demonstrated that inhibiting the action of 5-HT_2C_ receptors could attenuate pain. In a study by Baptista-de-souza, multiple injections of oxaliplatin significantly increased the mRNA levels of 5-HT_2C_ receptors in the spinal cord and PAG, and the selective 5-HT_2C_ receptor antagonist fluoxetine decreased the increased level of 5-HT_2C_ receptors in the spinal cord, but not in the PAG. In contrast to the results of Baptista-de-Souza and Chenaf, some studies have reported that intrathecal administration of various 5-HT_2C_ receptor agonists such as 6-chloro-2-(1-piperazinyl)-pyrazine, 1-(*m*-chlorophenyl)-piperazine, or 1-(*m*-trifluoromethylphenyl)-piperazine could significantly decrease neuropathic pain in rodents [108,109,110]. These results suggest that the role of spinal 5-HT_2C_ receptors may differ between pain models (i.e., spinal cord injury [SCI] vs. oxaliplatin). In addition, it should also be considered that the route of administration was different (intrathecal vs. intravenous and subcutaneous). Further studies that directly inject 5-HT_2C_ receptor agonists or antagonists into the spinal cord of oxaliplatin-induced pain rats need to be conducted to clarify the role of spinal 5-HT_2C_ receptors.

In contrast to 5-HT_2C_ receptors, activation of 5-HT_2A_ receptors was shown to alleviate pain, as spinal 5-HT_2A_ receptor antagonist pretreatment significantly blocked the analgesic effect of neurotropin. Taken together, these results demonstrate that 5-HT_2A_ receptors promote antinociception, whereas 5-HT_2C_ receptors play a pain-enhancing role in oxaliplatin-induced pain. Although future studies are needed to clarify the role of 5-HT_2A_ and 5-HT_2C_ receptor in oxaliplatin-induced pain, the function of these receptors may be different as 5-HT_2A_ receptors are known to be expressed in spinal inhibitory interneurons and have an antinociceptive role [111,112], whereas the activation of spinal 5-HT_2C_ receptors were reported to excites neurons [113,114,115], and its distribution in the spinal cord was demonstrated to be compatible with a pronociceptive role of 5- HT in the dorsal horn [116].

Furthermore, published reports of included studies suggested that spinal 5-HT_3_ receptors are involved in the attenuation of oxaliplatin-induced neuropathic pain. 5-HT_3_ receptors are known to be present in the superficial laminae of the spinal dorsal horn at the terminals of myelinated and unmyelinated primary afferent fibers. As mentioned previously, they are also known to mediate the release of GABA, but not glycine or glutamate [117,118]. In contrast to the analgesic role in oxaliplatin-induced neuropathy, in humans suffering from chronic neuropathic pain and SCI-induced neuropathic pain model mice, treatment with ondansetron, a 5-HT_3_ receptor antagonist, has been reported to produce a robust and long-term reduction in allodynia [119,120]. However, in our review, no studies have reported changes in behavioral response following administration of the 5-HT_3_ receptor antagonist. Although it is difficult to clarify the reasons for this inconsistency, it may be due to differences in the antagonists used (ondansetron vs. MDL-72222). It should be noted that ondansetron, along with its 5-HT_3_ receptor antagonistic effects, has been reported to act as a local anesthetic by blocking sodium channels [121,122].

In conclusion, our review demonstrates that 5-HT is not involved in the development of oxaliplatin-induced allodynia, but modulation of 5-HT may help attenuate allodynia. Furthermore, the results suggest that increasing the activity of the spinal 5-HT_1A_, 5-HT_2A_, and 5-HT_3_ receptors and decreasing the action of the spinal 5-HT_2C_ and 5-HT_6_ receptors may help to inhibit pain. However, more constructively designed experiments that use receptor knockout and selective agonists and antagonists should be conducted to deduce any firm conclusions. Considering that oxaliplatin is one of the most widely used anti-cancer agents and no optimal treatment for oxaliplatin-induced pain exists, our efforts to clarify the role of the serotonergic system may help other researchers to find an optimal drug to alleviate the suffering of patients with chemotherapy-induced peripheral neuropathic pain.

## Figures and Tables

**Table 1:** Involvement of 5-HT in oxaliplatin-induced neuropathic pain.

Authors	Strain	Oxaliplatin	Treatments	Findings
Masuguchi et al. (2014) [54]	SD rat	32 mg/kg, i.p., 8 times	Neurotropin (p.o., 50, 100, 200 NU/kg)	Depletion of 5-HT by PCPA (100 mg/kg, i.p.) did not affect the development of oxaliplatin-induced cold hyperalgesia and mechanical allodynia. Depletion of 5-HT abolished the analgesic effect of neurotropin (200 NU/kg) against both cold hyperalgesia and mechanical allodynia.
Lee et al. (2014) [27]	SD rat	6 mg/kg, i.p., single	BVA (s.c., 0.25 mg/kg)	Depletion of 5-HT by PCPA (150 mg/kg, i.p.) did not affect the development of oxaliplatin-induced cold allodynia. Depletion of 5-HT completely abolished the analgesic effect of BVA against oxaliplatin-induced cold allodynia. Spinal 5-HT level was upregulated after BVA treatment, whereas PCPA inhibited its increase.
Hache et al. (2014) [55]	C57BL/6J mouse	28 mg/kg, i.p., 4 times	Escitalopram (SSRI, s.c., 4 mg/kg), Venlafaxine (SNRI, s.c., 16 mg/kg), Indatraline (TRI, s.c., 3 mg/kg), NS18283 (TRI, s.c., 10 mg/kg)	Escitalopram, indatraline, and NS18283 suppressed mechanical allodynia induced by oxaliplatin. Venlafaxine, indatraline, and NS18283 showed significant efficacy in thermal preference test. Only indatraline increased latency on cold plate test. Extracellular 5-HT levels at ACC was significantly upregulated after escitalopram (215.7 ± 16.5% vs. 86.19 ± 7.0%), venlafaxine (283.8 ± 34.2% vs. 110.4 ± 3.4%), indatraline (194.6 ± 14.4% vs. 104.2 ± 8.03%), and NS18283 (196.0 ± 36.9% vs. 84.9 ± 11.6%) administration compared with vehicle treated group.
Li et al. (2015) [29]	SD rat	6 mg/kg, i.p., single	bvPLA_2_ (i.p., 0.2 mg/kg)	Depletion of 5-HT by PCPA (150 mg/kg, i.p.) did not affect the development of oxaliplatin-induced cold and mechanical allodynia. Depletion of 5-HT failed to block the analgesic effect of bvPLA_2_ against oxaliplatin-induced allodynia.
Li et al. (2019) [28]	C57BL/6 mouse	6 mg/kg, i.p., single	Venlafaxine (SNRI, i.p., 10, 40, 60 mg/kg)	Depletion of 5-HT by PCPA (150 mg/kg, i.p.) did not affect the development of oxaliplatin-induced cold and mechanical allodynia. Venlafaxine attenuated oxaliplatin-induced cold and mechanical allodynia. Depletion of 5-HT abolished the analgesic effect of venlafaxine (40 mg/kg) on mechanical but not cold allodynia.

Abbreviations: 5-HT; serotonin, ACC; anterior cingulate cortex, BVA; bee venom acupuncture, bvPLA_2_; bee venom phospholipase 2, i.p.; intraperitoneal, NU/kg; neurotropin units/kg, PCPA; para-chlorophenylalanine, p.o.; per os, s.c.; subcutaneous, SD rats; Sprague Dawley rats, SNRI; serotonin and norepinephrine reuptake inhibitor, SSRI; selective serotonin reuptake inhibitor, TRI; triple reuptake inhibitor.

**Table 2:** The role of 5-HT receptors in oxaliplatin-induced neuropathic pain.

Authors	Strain	Oxaliplatin	Treatments	Findings		
				Behavioral Changes	Mechanisms (↑: Increase, ↓: Decrease, -: Non Significant)	
Andoh et al. (2013) [62]	C57BL/6NCr mouse	3 mg/kg, i.p., single	Xaliproden (Selective 5-HT_1A_ receptor agonist, p.o., 0.3, 1, 3 mg/kg)	Fail to suppress mechanical allodynia	Oxaliplatin	mRNA of 5-HT_1A_R (DRG-, SC↑)
					Xaliproden	Tibial nerve firing↓
Masuguchi et al. (2014) [54]	SD rat	32 mg/kg, i.p., 8 times	Neurotropin (p.o., 50, 100, 200 NU/kg)	Inhibit cold hyperalgesia and mechanical allodynia	5-HT_2A_R (Ketanserin) 5-HT_3_R (MDL-72222) Antagonist (i.t.)	Effect blocked
					5-HT_1A_R (WAY100635) antagonist (i.t.)	Effect blocked (only cold allodynia)
					Pertussis toxin (Gi inhibitor, i.t.)	Effect blocked
Baptista-de-Souza et al. (2014) [67]	SD rat	36 mg/kg, i.p., 15 times	Fluoxetine (SSRI and 5-HT_2C_ receptor antagonist, s.c., 20 mg/kg)	Increase the paw pressure and licking latency Decrease withdrawal threshold	Oxaliplatin	mRNA of 5-HT_2C_R (SC and PAG↑, AMY↓, RVM-)
						Protein of 5-HT_2C_R (SC and PAG↑, RVM, AMY-)
					Fluoxetine	mRNA of 5-HT_2C_R (SC↓, AMY↑, RVM & PAG-)
						Protein of 5-HT_2C_R (SC↓, PAG and AMY↑, RVM-)
Lee et al. (2014) [27]	SD rat	6 mg/kg, i.p., single	BVA (s.c., 0.25 mg/kg)	Inhibit cold allodynia	5-HT_1/2_R (Methysergide) 5-HT_3_R (MDL-72222) antagonist (i.p.)	Effect blocked
					5-HT_3_R (MDL-72222) antagonist (i.t.)	Effect blocked
					5-HT_1A_R (NAN-190), 5-HT_2A_R (Ketanserin) antagonists (i.t.)	Failed to block
Kim et al. (2016) [25]	C57BL/6 mouse	6 mg/kg, i.p., single	BVA and Morphine (s.c., 1 mg/kg and 2 mg/kg, respectively)	Inhibit cold and mechanical allodynia	5-HT_3_R (MDL-72222) antagonist (i.t.)	Effect blocked
Lee et al. (2016) [26]	SD rat	6 mg/kg, i.p., single	EA (2 Hz, 20 min)	Inhibit cold allodynia	5-HT_3_R (MDL-72222) antagonist (i.t.)	Effect blocked
					5-HT_1A_R (NAN-190), 5-HT_2A_R (Ketanserin) antagonists (i.t.)	Failed to block
Andoh et al. (2016) [63]	C57BL/6NCr mouse	3 mg/kg, i.p., single	Xaliproden or Tandospirone (5-HT_1A_ receptor agonist, p.o., 0.3, 1, 3 mg/kg)	Inhibit mechanical allodynia	Xaliproden Tandospirone	Mast cell migration↓ (Plantar skin)
Chenaf et al. (2017) [68]	SD rat	18 mg/kg, i.v., 9 times	Agomelatine (5-HT_2C_ receptor antagonist, i.p., 45 mg/kg)	Increase lowered TWL	-	-
Salat et al. (2017) [64]	CD-1 mouse	10 mg/kg, i.p., single	NLX-112 (5-HT_1A_ receptor agonist, i.p., 1.25, 2.5, 5 mg/kg)	Inhibit mechanical allodynia Fail to inhibit cold allodynia	-	-
Rapacz et al. (2018) [65]	CD-1 mouse	10 mg/kg, i.p., single	3,3-diphenyl-propionamides (JOA 122 (3p), i.p., 1, 10, 30 mg/kg)	Inhibit mechanical allodynia	5-HT_1A_R	Binding affinity: 223.0 ± 4.5 nM K_i_ ± SEM
					5-HT_2A_R	Binding affinity: >5000 nM K_i_ ± SEM
					5-HT_3_R	-
Panczyk et al. (2018) [66]	CD-1 mouse	10 mg/kg, i.p., single	1-[3-(2,4,6-trimethylphenoxy)propyl]-4-(4-methoxyphenyl)piperazine dihydrochloride (Compound 3, 5-HT_1A_ receptor antagonist, i.p., 30 mg/kg)	Fail to inhibit mechanical allodynia	5-HT_1A_R antagonism	Binding affinity: 146.0 ± 28.4 nM K_i_ ± SEM
Li et al. (2019) [28]	C57BL/6 mouse	6 mg/kg, i.p., single	Venlafaxine (SNRI, i.p., 10, 40, 60 mg/kg)	Inhibit cold and mechanical allodynia	5-HT_3_R (MDL-72222) antagonist (i.t.)	Effect blocked
					5-HT_1/2_R (Methysergide) antagonist (i.t.)	Failed to block
Martin et al. (2020) [69]	SD rat	6 mg/kg, i.p., single	SB258585 (5-HT_6_ receptor inverse agonist, i.p., 1, 5 μmol/kg) PZ-1388 (5-HT_6_ receptor antagonist, i.p., 5, 25 μmol/kg)	Inhibit cold and mechanical pain behaviors	Tat-VEPE (reducing 5-HT_6_R-mTOR interaction)	Effect blocked
Lee et al. (2021) [46]	C57BL/6 mouse	6 mg/kg, i.p., single	Water extract of *Z. officinale* (p.o., 100, 300, 500 mg/kg)	Inhibit cold and mechanical allodynia	Oxaliplatin	mRNA of 5-HT_1A_R (SC↓)
					*Z. officinale*	mRNA of 5-HT_1A_R (SC↑)
					5-HT_1/2_R (Methysergide), 5-HT_1A_R (NAN-190) antagonist (i.t.)	Effect blocked

Abbreviations: 5-HT; serotonin, 5-HTR; serotonin receptor, AMY; amygdala, BVA; bee venom acupuncture, DRG; dorsal root ganglion, EA; electroacupuncture, i.v.; intravenous, i.p.; intraperitoneal, i.t.; intrathecal, mRNA; messenger RNA, NU/kg; neurotropin units/kg, PAG; periaqueductal gray, p.o.; per os, RVM; rostral ventromedial medulla, SC; spinal cord, SD rats; Sprague Dawley rats, SEM; standard error of the mean, SNRI; serotonin and noradrenalin reuptake inhibitor, SSRI; selective serotonin reuptake inhibitor, Tat-VEPE; fusion of sequence of amino acid (FFVTDSVEPE) to transduction domain of HIV Tat protein, TWL; tail withdrawal latency, *Z. officinale*; *Zingiber officinale.*
